# Evaluation of accuracy and reliability of OneCeph digital cephalometric analysis in comparison with manual cephalometric analysis—a cross-sectional study

**DOI:** 10.1038/s41405-021-00077-2

**Published:** 2021-06-17

**Authors:** Akshay Mohan, Arvind Sivakumar, Prasad Nalabothu

**Affiliations:** 1 Department of Orthodontics and Dentofacial Orthopedics, Saveetha Dental College and Hospitals, Saveetha Institute of Medical and Technical Sciences, Saveetha University, Chennai, India; 2 Department of Oral and Craniomaxillofacial Surgery, University Hospital Basel, Basel, Switzerland; 3 Department of Paediatric Oral Health and Orthodontics, University Center for Dental Medicine, UZB Basel, Switzerland

**Keywords:** Orthodontics, Dental radiology

## Abstract

**Introduction:**

Lateral cephalometric analysis continues to be one of the gold standard diagnostic aids in orthodontics, with various software available to enhance this.

**Aim:**

This study was done to evaluate the accuracy and reliability of linear and angular measurements obtained from OneCeph digital cephalometric tracing and manual tracings in lateral cephalometry.

**Methodology:**

This is a cross-sectional study done on twenty pre-treatment lateral cephalometric radiographs of subjects who reported to the postgraduate orthodontic clinic for orthodontic treatment over one month. Cephalometric tracings were done using OneCeph digital software and manual tracing method to evaluate nine parameters of Steiner’s cephalometric analysis. An Independent T-sample test was done between the mean values of manual and OneCeph tracing. Intra operator reliability was evaluated by paired *T*-test after a week.

**Results:**

No significant statistical difference was observed as the *p*-value was greater than 0.05 for all the parameters in the two groups.

**Conclusion:**

The reliability and accuracy of OneCeph software application was found to be at par with manual cephalometric tracing

## Introduction

Mobile phones have revolutionized our way of life and have become a part of our day-to-day life. From being used for the simple purpose of communication to currently being used for a wide range of purposes including finance, entertainment, education, and medicine; they have undergone a rapid metamorphosis. Over the past few years, mobile phones have rapidly changed how we treat our patients and hence aptly named smartphones. In orthodontics, smartphone apps are currently used for patient education, diagnosis, and treatment planning.^1,2^

The assessment of craniofacial structure forms an integral part of the orthodontic diagnosis. From the time lateral cephalogram was invented, lateral cephalometric analysis continues to be one of the gold standard diagnostic aids in orthodontics. Manual cephalometric analysis consumes valuable time due to the tedious procedures associated with it. Various cephalometric software is currently available in the market, which is easy to use and saves time.^3–10^ These software are expensive and would require a laptop or a desktop which makes it laborious and less accessible. Practitioners in most developing and underdeveloped countries find it difficult to afford such software.

The word “mobile phone” implies the advantage of accessibility and mobility on the go. Mobile cephalometric software app which is readily accessible through our smartphones is the need of the hour. One such app is the OneCeph (version beta 1.1, NXS, Hyderabad, India) which is free to use app available on the Android play store.^11^ In this study, we compared the accuracy and reliability of cephalometric measurements made using the OneCeph app against the conventional manual tracing.

## Materials and methods

This was a cross-sectional study that was carried out on pre-treatment lateral cephalometric radiographs collected from subjects who reported to the postgraduate orthodontic clinic for orthodontic treatment over a period of one month. This study design was approved by the institutional ethical committee (SDC/SIHEC/2020/DIASDATA/0619-0320). Orthodontic patients who reported to the hospital for orthodontic treatment having good quality lateral cephalograms taken with patients oriented in their natural head position. Patients with gross asymmetry, syndromes, radiographs with poor quality, faulty head positions, or any other conditions which make it difficult to identify the landmarks were excluded from the study. Sixty radiographs were collected over a period of one month based on inclusion and exclusion criteria. The selected radiographs were numbered from one to fifty based on chronological order. We had decided the sample size of twenty based on the previous studies that were done to assess the reproducibility and reliability of digital cephalometric software.^8,12^ A total of twenty radiographs were randomly selected for the study from the fifty radiographs using the software random.org, an online tool for randomization. All the patients were within the age group 18–32 years with a mean age of 22.4 ± 3 years.

All lateral cephalograms were taken using the same Papaya Dental OPG cephalostat (Genoray America Inc, Papaya) with 83 kVp voltage, 10 mA current, and exposure of 8 s. The radiographs were obtained in the JPG image format. For manual tracing, high-quality printouts of digital cephalograms were produced. Landmark identification is a major challenge in reducing errors. Previous studies have revealed that inconsistency in landmark identification is an important source of error in conventional cephalometry.^13,14^ All radiographs were manually and digitally traced and analyzed by a final year postgraduate student under the supervision of an experienced Orthodontist. To avoid errors due to fatigue, not more than four cephalograms were traced per day.

### Manual tracing

Manual tracings were carried out on an illuminated view box in a dark room. The tracing was done on a sheet of fine grade 36 µm matte acetate tracing paper taped over the X-ray printout and hand traced using a 0.3 mm lead pencil. Linear and angular measurements of Steiner’s cephalometric analysis were measured to the nearest 0.5 mm and 0.5° respectively.^15^

### Digital tracing

**Fig. 1:**
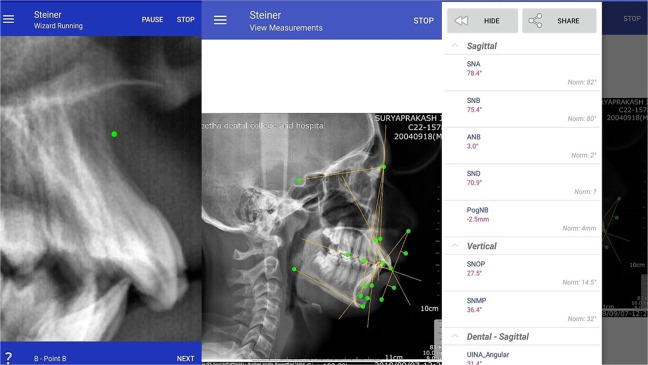
Digital tracing with OneCeph software. Digital tracing of cephalometric points and analysis done using the OneCeph software.

For digital cephalometric measurements, digital images of selected cephalograms in JPG format were imported to the OneCeph (Google Play Store, Google Inc, Mountain View, Calif) application on an android smartphone (Samsung S10 plus Smartphone, Samsung Telecommunications, Suwon, South Korea). After calibration of the images based on the calibration scale, skeletal and dental landmarks for Steiner’s analysis were identified by the same operator on digital images using a stylus (Fig. 1). After completion of landmark plotting, linear and angular measurements of Steiner’s analysis were obtained from the OneCeph application.^15^ All cephalometric measurements observed were entered into the Excel spreadsheet.

### Statistical analysis

All statistical analysis was carried out using Statistical Package for Social Sciences version 20.0 (SPSS Inc., Chicago, IL, USA). An independent sample *T*-test was done to compare the mean values of each parameter between manual tracing and OneCeph tracing keeping the level of significance at 95%. After a week of completing the initial measurements, five radiographs were randomly selected out of twenty previously selected radiographs and the measurements were repeated using OneCeph software and manual tracing to check for intra-operator errors using the paired *T*-test.

## Results

Mean, standard deviation, standard error, and the mean difference in the measurements were compared between the manual tracing and OneCeph tracing (Table 1). Independent sample *T*-test was done between the mean values of manual tracing and OneCeph tracing showed no statistically significant difference in all the parameters observed between the groups. The Intra operator reliability was assessed using a paired *T*-test and was estimated to be 0.93.

**Table 1:** The bar graph represents the mean, standard deviation, and standard error of manual tracing and OneCeph tracing for a confidence interval of 95% for the parameters of Steiner’s analysis.

	Manual tracing			One Ceph tracing			Significance
	Mean	SD	SE	Mean	SD	SE	
SNA angle,°	84.61	4.91	1.09	83.68	4.69	1.04	0.977
SNB angle,°	80.11	5.40	1.20	79.53	5.14	1.15	0.898
Mandibular plane angle,°	31.68	7.44	1.66	30.16	9.17	2.05	0.777
ANB angle,°	4.42	2.24	0.50	5.08	4.82	1.07	0.252
Upper incisor to NA, mm	7.70	3.44	0.76	8.93	2.82	0.63	0.432
Upper incisor to NA angle,°	31.32	7.55	1.69	30.95	8.79	1.96	0.602
Lower incisor to NB, mm	8.70	3.01	0.67	10.14	5.79	1.29	0.178
Lower incisor to NB angle,°	35.18	5.91	1.32	35.23	6.56	1.46	0.405
Occlusal plane angle,°	11.97	5.95	1.33	18.35	6.16	1.37	0.811

Statistical difference was not observed among the two groups (Independent sample *T*-test; *p* value > 0.05). The unit of measurement for the angle is the degree (°), and linear measurements are in millimeters (mm).

## Discussion

OneCeph is one of the few easily available software which can be downloaded from the Google Play store app in any of the current smartphones which run on the Android operating systems. The reliability and reproducibility of this newly launched software have not been compared with the conventional manual tracing. Hence in our study, we compared the accuracy of cephalometric analysis done using both OneCeph software and manual tracing to verify the accuracy of the software. Steiner’s analysis was chosen for this study because it is one of the most widely used cephalometric analyses which has both angular and linear measurements as well as skeletal and dental parameters.^15^ It is observed that when the hard copies of the radiographs are converted into soft copies the accuracy of scanning plays a vital role as it can lead to distortion.^16^ Therefore in our study the previous records which were mostly hard copies of cephalograms were not included in the study to avoid any errors in scanning.

The comparison of the mean measurements for all the parameters of Steiner’s analysis between the groups showed that there was no significant difference between both the techniques. The intra-operator reliability assessed by paired *T*-test showed that there was high reliability among the measurements.

Similar studies have been done for desktop software like Dolphin^®^, NemoCeph, Vistadent^TM^, Quick Ceph, AOCeph^TM^, FACAD^®^, and AutoCEPH^©^. The authors have claimed that the accuracy and reliability of this software are similar to the manual cephalometric tracing and therefore can be used as an aid in diagnosing, planning, monitoring, and evaluating orthodontic treatment both in clinical and research settings.^3–10^ However, the disadvantages of desktop cephalometric software are that it can only be used on a desktop or a laptop, expensive, and require an internet connection.

In recent years, much cephalometric software like Smile-Ceph, Ceph Ninja, and Smart Ceph Pro apps have been launched in the market, which can be used on tablets and smartphones. Few of the studies have found that these mobile digital cephalometric software and applications were accurate and can be used as an alternative to manual tracing.^17,18^ A study by Gorracci et.al showed good reliability for all cephalometric measurements obtained with an iPad-based software Smile-Ceph, desktop software NemoCeph and manual tracing.^12^ One of the disadvantages of this software is that it can be accessed on an iPad tablet and IOS devices only.

OneCeph is one such mobile software that is easy to use, quick, and easily dispensable, and user-friendly as it is operated by Android mobile phones.^11^ The software is versatile as it can be used to do most of the conventional as well as contemporary cephalometric analysis. OneCeph can work on a smartphone even without an internet connection; hence can be used in doing studies in rural centers with less accessibility to the internet. However, this software is currently available only in the android play store and not available in other operating systems like Windows, IOS, etc. Android smartphones are widely used in developing countries as it is easily available and affordable. Hence, OneCeph software can be conveniently used by dental practitioners and dental students serving in primary health care centers in rural areas. Since this software can do analysis only on 2D images hence the disadvantages of all the 2D analysis apply to this software as well. An integrated approach of diagnosis, treatment planning using smartphone cephalometric analysis software will be a valuable tool in rural villages in developing countries with little access to specialized oral health care services, where there is an immense need for orthodontic treatment, orthognathic surgery, cleft, and craniofacial deformity management. With the recent advent of the COVID-19 pandemic, orthodontic expertise can be shared with the general dental practitioners serving in rural dental clinics via teleconferencing and can successfully enhance the timely orthodontic intervention for patients with urgent need.

The influence of technology has become very prominent and has emerged as a critical part of medical and dental education, clinical research, diagnosis, and treatment planning. The widespread use of dentistry-related smartphone apps by students and practitioners to supplement their learning and clinical practice is a testimony of technological advancement. These apps can easily be integrated into the digital workflow thus improving patient management efficiency. Moreover, the cephalometric results obtained from the OneCeph app can be stored, used, and retrieved as per the need saving a lot of office space that would otherwise be consumed in the storage of records. This study used variables from an extensively practiced cephalometric analysis to simulate a real-life experience and to test uniformly the performance of the app.

### Limitations

This OneCeph application needs to be compared with other applications available in the market to find the difference in their accuracy, performance, and efficiency.

## Conclusion

The reliability and accuracy of the OneCeph software application were at par with manual tracing. OneCeph is a simple, reliable, accurate alternative to manual tracing which can be easily accessed on a smartphone without an internet connection thereby saving clinical time and armamentarium.
